# Metastasis patterns and prognosis in young gastric cancer patients: A propensity score‑matched SEER database analysis

**DOI:** 10.1371/journal.pone.0301834

**Published:** 2024-04-09

**Authors:** Hong Zhang, Xia Cheng, Wenqin Guo, Cheng Zheng, Yue Zhang, Xiaoying Jing, Hui Qiao

**Affiliations:** 1 School of Public Health and Management, Ningxia Medical University, Yinchuan, Ningxia, China; 2 Health Management Center, People’s Hospital of Ningxia Hui Autonomous Region, Yinchuan, Ningxia, China; 3 Clinical Medical Research Center, People’s Hospital of Ningxia Hui Autonomous Region, Yinchuan, Ningxia, China; 4 School of Nursing, Ningxia Medical University, Yinchuan, Ningxia, China; 5 School of Medicine, Wuhan University of Science and Technology, Wuhan, Hubei, China; E-Da Cancer Hospital, TAIWAN

## Abstract

**Background:**

Whether young patients with metastatic gastric cancer (GC) had distinct metastasis patterns and survival outcomes from older patients remains controversial. The aim of the present study was to explore the metastasis patterns and prognostic factors in young patients and evaluate the survival outcome in comparison to their older counterparts.

**Materials and methods:**

We identified patients with metastatic GC in the surveillance, epidemiology, and end results (SEER) database from 2010 to 2015. The patients were divided into two groups based on age at diagnosis: younger (≤40 years old) and older (>40 years old). We employed the chi‐squared test to compare the clinicopathological characteristics between the two age groups. Furthermore, we conducted survival analyses using Kaplan–Meier and Cox regression analyses. To balance disparities in baseline characteristics, we employed propensity score matching (PSM).

**Results:**

We identified 5,580 metastatic GC patients from the SEER database, with 237 (4.2%) classified as younger and 5343 (95.8%) as older patients. A total of 237 pairs of patients were generated after adjustment by PSM. Patients in the younger group exhibited a higher proportion of bone-only metastases and a lower proportion of liver-only metastases compared with patients in the older group. Multivariate Cox regression analysis demonstrated that youth was an independent protective factor for overall survival (OS) before and after PSM, but not for gastric cancer-specific survival (GCSS). Among the younger group, patients with liver-only metastasis demonstrated the best prognosis, whereas patients with lung-only metastasis exhibited significantly worse survival outcomes compared with liver-only metastases, even comparable to that of bone metastasis.

**Conclusions:**

Compared with the older group, the metastatic GC patients in the younger group exhibited more aggressive tumors but better prognoses. The metastasis pattern and its effect on the prognosis of GC varied by age group.

## Introduction

In 2020, gastric cancer (GC) accounted for 1,089,103 cases globally, making it the fifth most prevalent cancer, and ranked the fourth leading cause of cancer-related deaths, accounting for 768,793 deaths [1]. Typically, the incidence of GC is higher among patients aged 50–70 years [2], whereas young patients have a relatively lower incidence. GC in patients less than 40 years of age accounts for approximately 4.6%–6.2% [3–5]. In many parts of the world, the incidences and mortality of GC have been decreasing for several decades due to economic development and medical screening [6]. However, a stable or even slightly increasing trend of GC incidences and mortalities has been reported in young patients [7–9]. Furthermore, since younger patients often represent highly productive individuals in society, these deaths significantly impact not only the patients but also their families and society [10,11]. Thus, conducting further research on GC in younger patients is imperative.

Metastasis plays a crucial role in the mortality of patients with GC. At diagnosis, about 35% of patients exhibit metastatic disease [12,13], and the median overall survival is often less than 1 year [14,15]. Several studies have indicated a high incidence of metastasis and poor prognosis in younger patients with GC [16–18]. However, some other recent studies have reported that the prognosis in younger patients with GC is comparable to or even better than that in older patients [19,20]. For patients with metastatic GC, the liver, lung, and bone are typical sites of distant metastasis [21]. Nevertheless, the metastasis pattern and its effect on the prognosis of GC in different age groups remains unclear.

In the present study, we examined the clinicopathological features, metastasis pattern, and prognosis of metastatic GC in younger patients based on the surveillance, epidemiology, and end results (SEER) database. In addition, we evaluate the survival outcome in comparison to their older counterparts using propensity score matching (PSM) analysis.

## Materials and methods

### Data collection

We extracted data from the SEER database, employing the SEER 17 Registries Database based on November 2021 submission (2000–2019 data set). The SEER database, which covers about 26.5% of the US population, is one of the largest population-based cancer registries in the world. As SEER is a publicly available database with deidentified data, the institutional review board approval and formal consent from patients were not required in this study. We have obtained permission to access the SEER database with the authorization code 25567-Nov2021.This study adhered to the World Medical Association’s Declaration of Helsinki for Ethical Human Research.

### Patients

We used the SEER*Stat version 8.3.6 software to identify patients with metastatic GC from the SEER registry database, including additional treatment data. The inclusion criteria were: 1) diagnosis between 2010 and 2015, 2) primary site at the stomach, and 3) M stage was based on the 7th edition of the American Joint Committee on Cancer (AJCC) staging system. The exclusion criteria were: 1) patients with unknown survival time, 2) patients who had died within 1 month after the initial diagnosis, 3) patients with an unknown metastatic site, and 4) patients diagnosed at the T0 stage. The flowchart of patient selection in this study is shown in S1 Fig.

### Study variables

We extracted variables from the SEER database, including age at diagnosis (reclassified as ≤40 years old and >40 years old), sex (female, male), race (white, black, and others (American Indian/AK Native, Asian/Pacific Islander), T stage (AJCC 7^th^ edition), N stage (AJCC 7^th^ edition), differentiation, primary site, site of distant metastasis (liver, bone, brain, lung), treatment modalities (surgery + chemo- and/or radio-therapy, chemo- and/or radio-therapy, no/unknown), vital status for individual patients, and survival time. Furthermore, we categorized the histologic subtype as well/moderately differentiated, poorly differentiated/undifferentiated, and unknown. We defined the primary site based on ICD-O-3 codes and reclassified it into five different parts: cardiac/fundus, body, antrum/pylorus, lesser and greater curvature, and others. The grouping of treatment modalities was based on records of surgery (Reason no cancer-direct surgery), chemotherapy (chemotherapy recode), and radiotherapy (radiation recode) in the SEER database. To evaluate survival outcomes, OS and gastric cancer-specific survival (GCSS) were defined as the primary endpoints. OS was computed from the date of diagnosis to the last follow-up or death from any cause, while GCSS was computed from the date of diagnosis to death from GC. For analysis, the age at disease diagnosis of 40 years was used as the cutoff point based on most previous literatures [22–25].

### Statistical analysis

The chi-square test was employed to compare the basic clinical characteristics of patients in the younger and older groups. To estimate OS and GCSS, we employed the Kaplan–Meier method and performed comparisons using the log-rank test. The multivariate Cox regression analysis models were employed to compute hazard ratios (HRs) and 95% confidence intervals (CIs) to assess the impact of different factors on OS and GCSS. To balance the effect of baseline characteristics on survival outcomes, we conducted 1:1 PSM with a caliper of 0.02 to match patients in the younger and older groups. The matching factors included sex, race, differentiation, primary site, site of distant metastasis, and treatment modalities. Additionally, we conducted a chi-square test and survival analysis using SPSS 21.0 (SPSS Inc., Microsoft, Chicago IL, USA). PSM was performed using Stata17 for Windows (Stata Press, 2019). We plotted survival curves using GraphPad Prism 6.01 (GraphPad Software Inc., CA, USA) and generated forest plots using R software (R Foundation). We set the level of significance at *P* < 0.05, and all statistical tests were two-sided.

## Results

### Patient characteristics

We extracted data from the SEER database consisting of 5,580 patients with metastatic GC diagnosed between 2010 and 2015. Among them, 237 (4.2%) patients were diagnosed at the age of ≤40 years old and 5,343 (95.8%) patients at >40 years old. We observed significant differences in characteristics in race, differentiation, primary site, treatment modalities, and metastatic sites between the two groups (*P* < 005). Younger patients with metastatic GC exhibited a lower proportion in the well and moderately differentiated grade (16.5% vs. 25.5%, *P*<0.05) and primary site at the cardiac/fundus (37.1% vs. 46.0%, *P*<0.05) compared with the older group. In terms of treatment, patients in the younger group were more likely to receive chemo- and/or radiotherapy (77.2% vs. 65.0%, *P*<0.05) (Table 1).

**Table 1:** Comparison of the clinical and pathological characteristics between two age groups before and after PSM.

Variables	Before PSM			After PSM		
	≤ 40 years old (n = 237)	> 40 years old (n = 5343)	*P*	≤ 40 years old (n = 237)	> 40 years old (n = 237)	*P*
Age						
Median	35	67		35	54	
Sex			0.291			0.563
Male	157 (66.2%)	3712 (69.5%)		157 (66.2%)	151 (63.7%)	
Female	80 (33.8%)	1631 (30.5%)		80 (33.8%)	86 (36.3%)	
Race			0.002			0.273
White	164 (69.2%)	3915 (73.3%)		164 (69.2%)	179 (75.5%)	
Black	26 (11.0%)	772 (14.4%)		26 (11.0%)	23 (9.7%)	
Other^a^/unknown	47 (19.8%)	656 (12.3%)		47 (19.8%)	35 (14.8%)	
T stage			0.211			0.570
T1/T2/T3	75 (31.6%)	1993 (37.3%)		75 (31.6%)	65 (27.4%)	
T4	45 (19.0%)	933 (17.5%)		45 (19.0%)	45 (19.0%)	
TX	117 (49.4%)	2417 (45.2%)		117 (49.4%)	127 (53.6%)	
N stage			0.656			0.643
N0	82 (34.6%)	1985 (37.2%)		82 (34.6%)	80 (33.8%)	
N1/N2/N3	113 (47.7%)	2502 (46.8%)		113 (47.7%)	107 (45.1%)	
NX	42 (17.7%)	856 (16.0%)		42 (17.7%)	50 (21.1%)	
Differentiation			0.007			0.898
Well/Moderately	39 (16.5%)	1363 (25.5%)		39 (16.5%)	41 (17.3%)	
Poorly/Undifferentiated	137 (57.8%)	2744 (51.4%)		137 (57.8%)	132 (55.7%)	
Unknown	61 (25.7%)	1236 (23.1%)		61 (25.7%)	64 (27.0%)	
Primary site			0.025			0.335
Cardiac/fundus	88 (37.1%)	2456 (46.0%)		88 (37.1%)	89 (37.6%)	
Body	28 (11.8%)	447 (8.4%)		28 (11.8%)	15 (6.3%)	
Antrum/pylorus	28 (11.8%)	723 (13.5%)		28 (11.8%)	32 (13.5%)	
Lesser/greater	25 (10.5%)	477 (8.9%)		25 (10.5%)	26 (11.0%)	
Other^b^	68 (28.7%)	1240 (23.2%)		68 (28.7%)	75 (31.6%)	
Treatment			<0.001			0.942
Surgery + chemo-and/or radio-therapy	25 (10.5%)	467 (8.7%)		25 (10.5%)	27 (11.4%)	
Chemo- and/or radio-therapy	183 (77.2%)	3471 (65.0%)		183 (77.2%)	180 (75.9%)	
No/Unknown	29 (12.2%)	1405 (26.3%)		29 (12.2%)	30 (12.7%)	
Metastatic sites			0.001			0.529
Liver only	116 (48.9%)	3122 (58.4%)		116 (48.9%)	127 (53.6%)	
Bone only	46 (19.4%)	569 (10.6%)		46 (19.4%)	45 (19.0%)	
Brain only	4 (1.7%)	71 (1.3%)		4 (1.7%)	1 (0.4%)	
Lung only	24 (10.1%)	485 (9.1%)		24 (10.1)	18 (7.6%)	
Multiple sites	47 (19.8%)	1096 (20.5%)		47 (19.8%)	46 (19.4%)	

Abbreviations: PSM, propensity score matching. a, American Indian/AK Native; Asian/Pacific Islander. b, overlapping/stomach.

### Relationship between age and metastasis patterns

**Fig 1:**
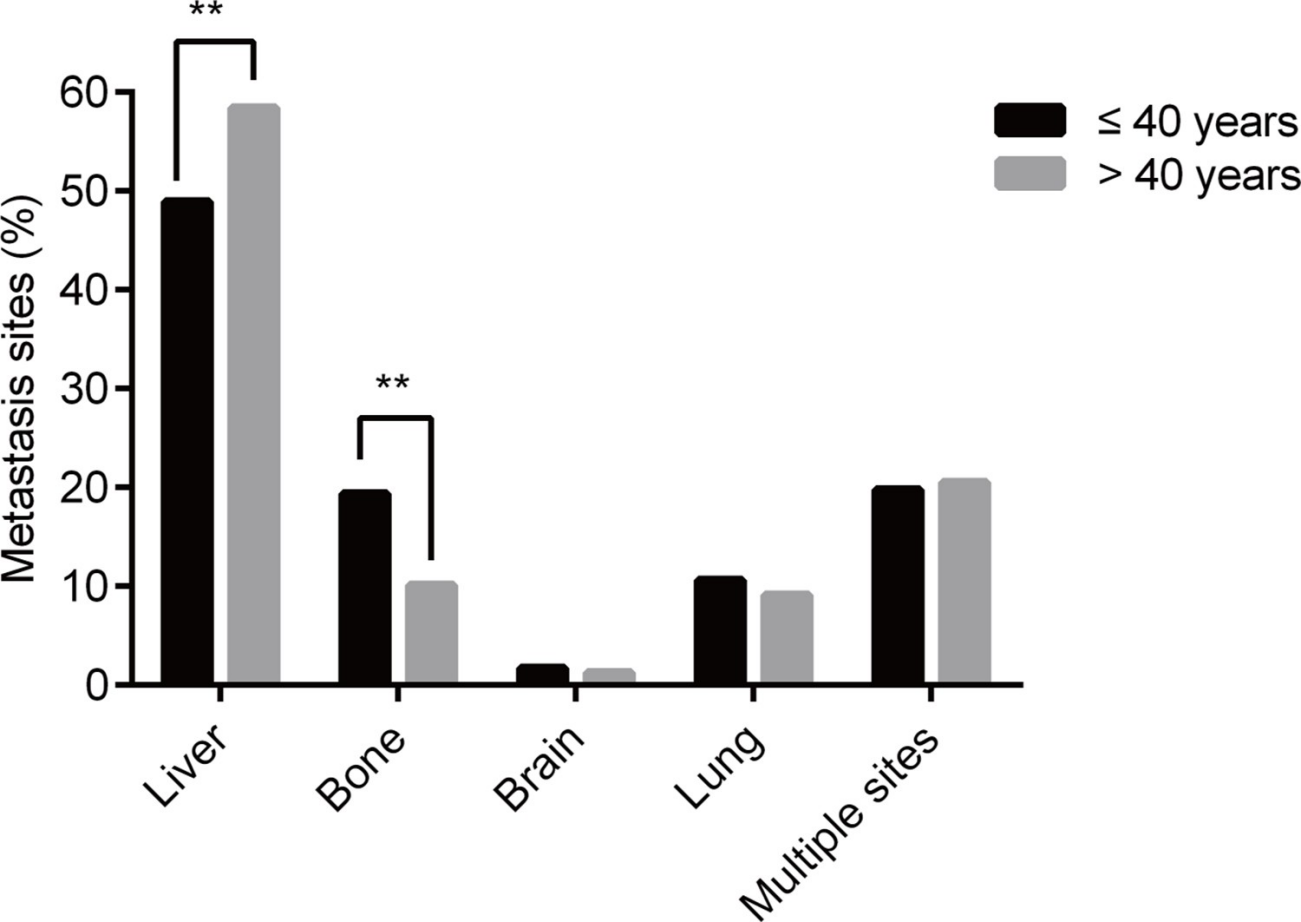
The metastasis patterns between two age groups (≤40 years versus >40 years). ** *P* < 0.001.

Among the study population, 4437 (79.5%) patients exhibited single-site metastasis, whereas 1143 patients (20.5%) had multiple-site metastasis. For single-site metastasis, the most common site was liver-only metastasis (n = 3238, 58.0%), followed by bone-only metastasis (n = 615, 11.0%), lung-only metastasis (n = 509, 9.1%), and brain-only metastasis (n = 75, 1.3%). For multisite metastasis, the most prevalent pattern was liver metastasis with lung metastasis (n = 691, *P* = 60.5%). Furthermore, it is noteworthy that the proportion of liver-only metastasis was substantially lower in the younger group (48.9% vs. 58.4%, *P* = 0.004) than in the older group, while the proportion of bone-only metastasis was higher in the younger group (19.4% vs. 10.6%, *P*<0.001). However, no substantial difference was observed in lung-only metastasis, brain-only metastasis, and multiple-site metastasis between the two groups. Fig 1 shows the detailed results.

### Impact of age on GC survival outcomes

**Fig 2:**
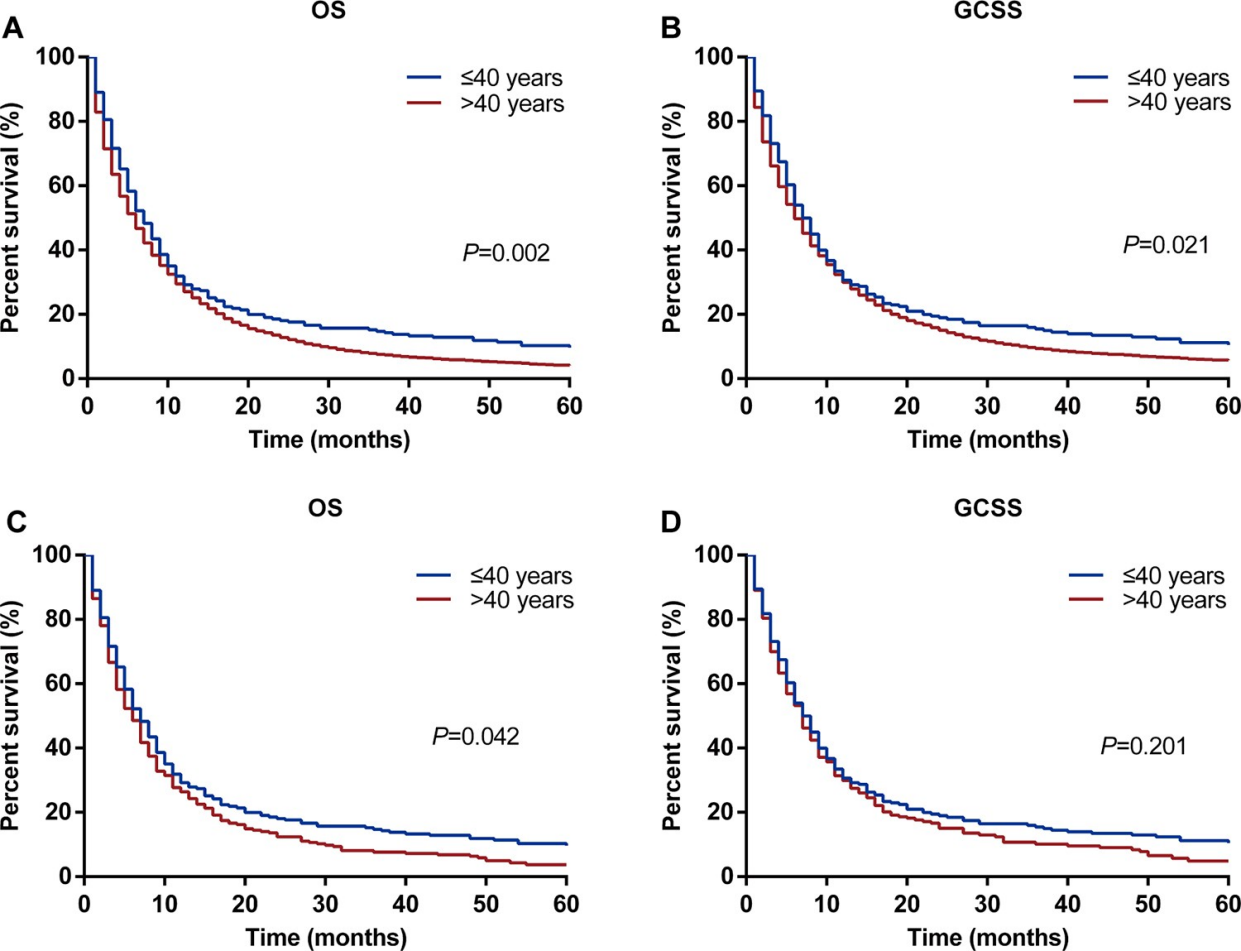
Kaplan–Meier survival curves of OS and GCSS by age groups before and after PSM. Kaplan–Meier survival curves of OS (A) and GCSS (B) by age groups (≤40 years versus >40 years) before PSM; Kaplan–Meier survival curves of OS (C) and GCSS (D) by age groups (≤40 years versus >40 years) after PSM.

Fig 2 illustrates the OS and GCSS curves stratified by age group. The younger group exhibited a significantly higher OS rate (11.8% vs. 4.4%; *P* = 0.002; Fig 2A) and GCSS rate (15.6% vs. 12.3%; *P* = 0.021; Fig 2B) compared with the older group. To balance disparities between the younger and older groups, PSM was conducted. Ultimately, 237 patients in the older group were selected to match 237 patients in the younger group. After PSM, balance in patient characteristics was achieved (*P* > 0.05, Table 1). Subsequently, the younger group still demonstrated a better OS (11.8% vs. 4.6%; *P* = 0.042; Fig 2C) than the older group. However, age at diagnosis showed no significant correlation with GCSS (15.6% vs. 14.3%; *P* = 0.201; Fig 2D).

**Fig 3:**
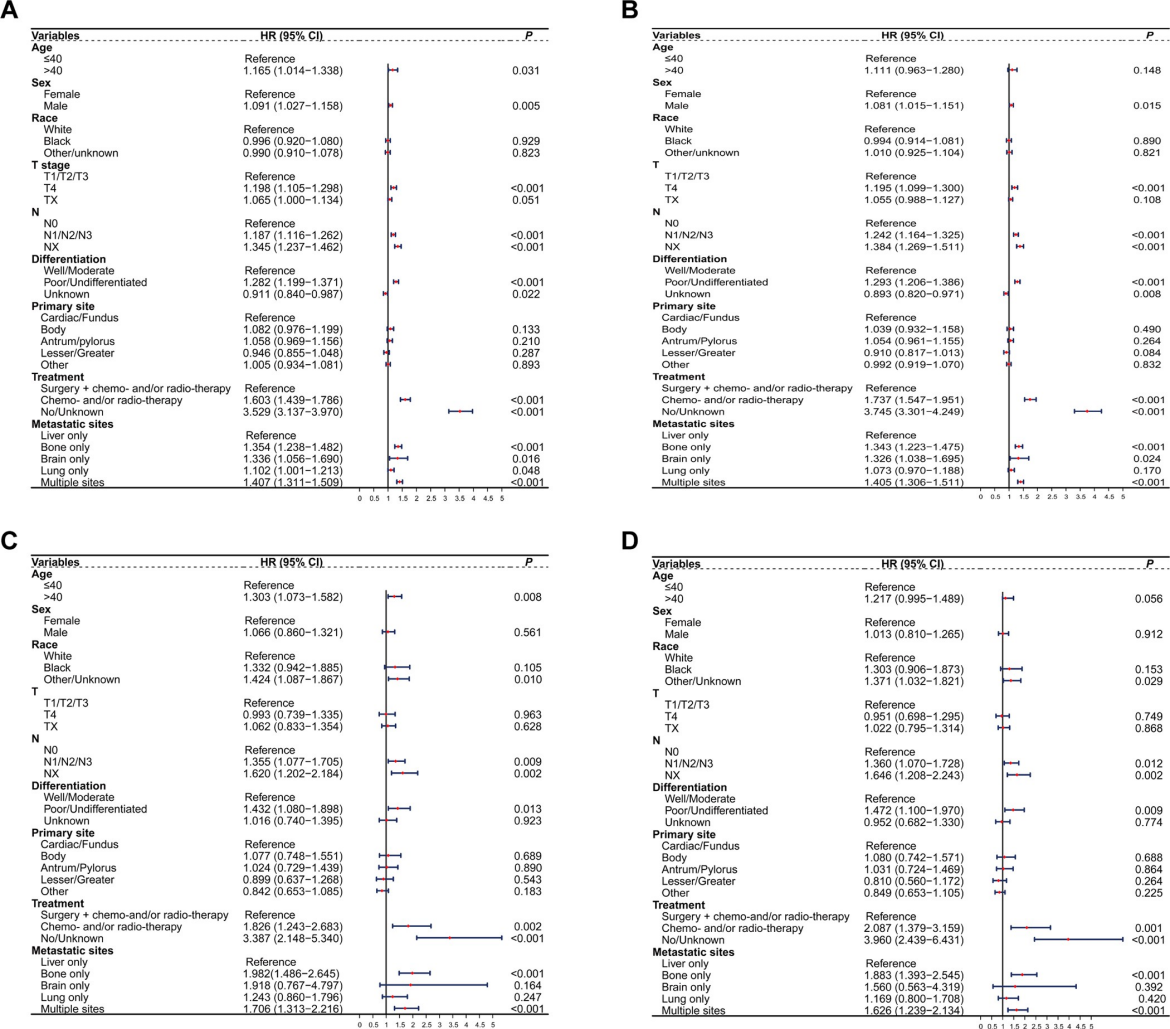
Forest plot of factors associated with OS and GCSS before and after PSM. Forest plot of factors associated with OS (A) and GCSS (B) before PSM; Forest plot of factors associated with OS (C) and GCSS (D) after PSM.

We incorporated all variables into the Cox regression model and identified significant associations between prognosis in patients with metastatic GC and age at diagnosis, sex, T stage, N stage, differentiation, treatment modalities, and metastasis sites (Fig 3A and 3B). Age at diagnosis ≤ 40 years old was an independent protective factor for OS (HR: 1.165; 95% CI 1.014–1.338; *P* = 0.031; Fig 3A) but not for GCSS (HR: 1.111; 95% CI 0.963–1.280; *P* = 0.148; Fig 3B). For the PSM-matched group, age at diagnosis ≤ 40 years old demonstrated an OS benefit (HR: 1.303; 95% CI 1.073–1.582; *P* = 0.008; Fig 3C) in multivariate analysis, whereas GCSS (HR: 1.217; 95% CI 0.995–1.489; *P* = 0.056; Fig 3D) did not. Furthermore, race, N stage, differentiation, treatment modalities, and metastasis sites were independent prognostic factors (Fig 3C and 3D). On evaluating the individual sites of metastasis, metastasis to the bone exhibited the strongest association with poor OS (HR:1.982, 95% CI 1.486–2.645, *P*<0.001) and GCSS (HR:1.883, 95% CI 1.393–2.545, *P*<0.001) among included sites.

**Fig 4:**
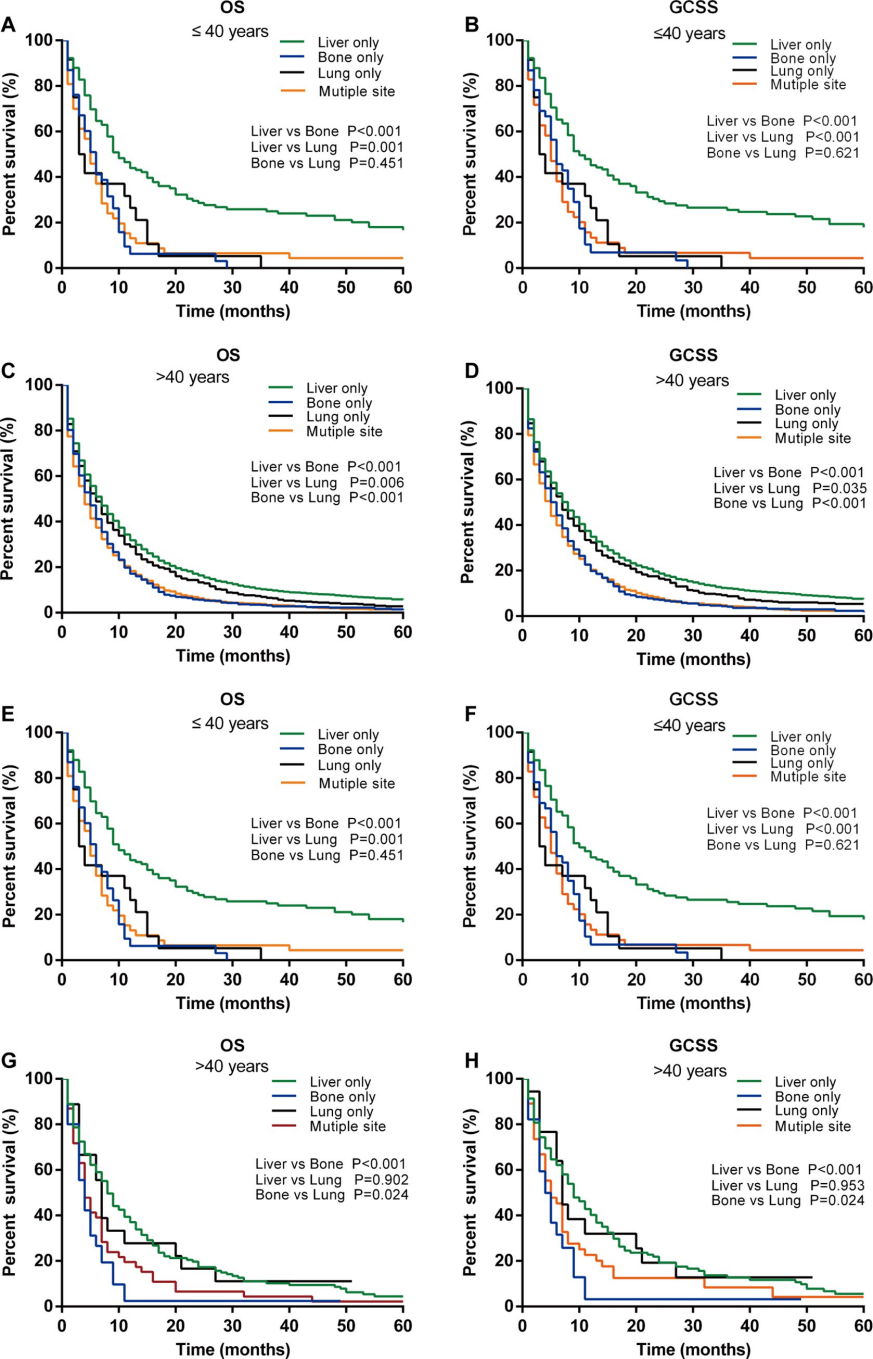
Kaplan–Meier survival curves of OS and GCSS according to metastasis sites. Kaplan–Meier survival curves of OS and GCSS according to metastasis sites in younger (≤40 years) groups before (A and B) and after (E and F) PSM; Kaplan–Meier survival curves of OS and GCSS according to metastasis sites in older (>40 years) groups before (C and D) and after (G and H) PSM.

After PSM, the analysis of the effect of metastasis site on prognosis in different age groups revealed that there was only one patient with brain-only metastasis in the older group. Patients with brain-only metastases were not used for further analysis because the small sample size may affect the accuracy of the results. In both the younger and older groups, patients with liver metastasis exhibited the most favorable OS and GCSS, followed by those with lung- and bone-only metastases (Fig 4A–4D). The findings were the same after PSM (Fig 4E–4H). In the younger group, patients with lung-only and bone-only metastases appeared to have similar OS and GCSS, and both are significantly worse than those with liver-only metastases. (Fig 4E and 4F). However, in the older group, the OS and GCSS of patients with lung-only metastasis is similar to that of patients with liver-only metastasis, both of which are significantly better than those with bone-only metastasis (Fig 4G and 4H).

## Discussion

Accurate assessment of the prognosis of metastasis GC patients in young individuals remains challenging due to its rarity. Moreover, the age cutoff value for young patients varies widely across the literature [8,26,27]. In this study, we employed data from the SEER database, which offers a large sample size to identify factors associated with patient survival. Based on age at diagnosis, we categorized metastatic GC patients into two groups. We found that patients in the younger group were more likely to have single bone metastasis, but less likely to have single liver metastasis compared with patients in the older group. The PSM and multivariate regression analysis demonstrated that age is an independent factor influencing the prognosis of metastatic GC. Younger patients with metastatic GC were associated with more favorable survival outcomes than older patients.

Previous studies have reported that GC patients diagnosed at younger ages are associated with poorly differentiated histology, advanced tumor stage at the time of diagnosis, and metastatic disease, indicating that these cases may be more aggressive [17,18,28]. Li et al. identified 11,299 GC patients with distant metastasis and discovered that young GC patients exhibited more poor or undifferentiated grades and a higher proportion of stage T3 and T4 [29]. Bergquist, J. R. et al. also observed that GC in young patients exhibited more aggressive features and were at a more advanced stage compared with those in older patients [8]. Consistent with the findings of previous studies, the GC lesions of patients in the younger group presented substantially more poor or undifferentiated grades and were more located in the middle third of the stomach compared with those of patients in the older group. Several factors might explain these more aggressive tumors in younger patients. These include delayed diagnosis because of low GC incidence and health screening systems for GC rarely considered in younger patients [30,31], or a higher proportion of H. pylori infection [32,33].

The literature lacks a comprehensive characterization of the metastatic patterns of GC. Riihimäki, M. et al. conducted a study on 7,759 patients with GC using Swedish registers and identified the liver as the most common metastasis site in 48% of patients [34]. Similarly, Chen et al. demonstrated a high incidence rate (35%) of liver metastases in GC patients in China. In this study, we discovered that liver metastasis was the most common pattern of distant metastases in the younger (48.9%) and older (58.4%) groups. The high incidence of liver metastasis in GC may be attributed to differences in the metastasis pathways and the popularity of inspection measures. Studies have demonstrated that GC cells spread to various organs through the portal vein, and the liver serves as the primary filter for these cells [35,36]. Furthermore, GC cases with early liver metastasis can be detected due to recent advancements in imaging techniques. Additionally, we compared the metastatic patterns between the two age groups and discovered a higher proportion of bone metastasis in the younger patients and a higher proportion of liver metastasis in the elderly patients. A prior SEER research identified that patients in the younger age group were more likely to have single bone metastasis [36]. This could be attributed to the more poorly differentiated tumors in young patients. Previous studies have demonstrated that poorly differentiated tumors are more likely to develop bone metastasis [37–39]. However, further exploration is required to understand the underlying molecular mechanisms.

In patients with metastatic GC, the impact of age on prognosis remains controversial. Tekesin, K. et al. discovered that younger age (≤40 years old) at disease diagnosis resulted in comparable survival outcomes compared with their old counterparts [40]. A retrospective study in the United States demonstrated that the median survival was shorter in younger patients compared with older patients, but stage-specific survival was similar [28]. Our results demonstrated that age at diagnosis is an independent prognostic factor for prognosis. Multivariate COX demonstrated that youth was an independent protective factor for OS but not for GCSS. Recently, a study in the German population also reported significantly better survival in younger patients [27]. The decrease in mortality in younger patients may be attributed to better physiologic function and fewer comorbidities. Furthermore, a therapy bias between younger and older groups cannot be ignored, with younger patients being more likely to receive treatment, especially those at advanced stages.

The relationship between metastatic patterns and survival outcomes yielded interesting results. Patients with liver-only metastasis demonstrated the longest OS compared with those with other metastases, whereas the bone metastasis-only group and the multisite metastases group had the poorest outcomes. In the older group, there were no significant differences in survival between lung-only vs. liver-only metastases. However, in the younger group, patients with lung-only metastases exhibited significantly worse survival outcomes compared with those with liver-only metastases, and even comparable to those with bone-only metastases. This observation may be associated with the lesions being poorly differentiated in younger GC patients. During the early stages of lung metastasis, there are no obvious clinical symptoms, but once diagnosed, the disease often develops into late stages and the metastases are always found bilaterally. Guo, Y. et al. revealed that the bilateral lung metastasis was an independent risk prognostic factor in GC patients with lung metastasis [41]. The specific mechanism requires further investigation.

The results of this study may provide information for clinical management and personalized treatment for GC by age. Kist, M. et al. reported that multimodal treatment or definitive radio-chemotherapy was applied more frequently in young patients than in older patients [27]. Zhao, B. et al. revealed that younger patients being more likely to receive chemotherapy [42]. Consistent with previous studies, our results found a higher rate of Chemo- and/or radio-therapy in younger patients. These findings are somewhat because of young patients presented more nonspecific symptoms and advanced tumors. It highlights on the one hand that rigorous diagnostic workup, such as early endoscopy, should be applied in younger GC patients. On the other hand, our data can impact clinical decision making as we found favorable outcomes in these younger patients even in the metastatic GC, and which implicating aggressive treatment is appropriate in the younger metastatic GC patient.

This study has some limitations. First, due to its retrospective nature, complete elimination of selection bias is not feasible, potentially impacting the external validity of the findings. Second, information was only available on metastasis to the liver, lung, bone, and brain, while other sites such as peritoneal metastases were not accounted for. Third, the SEER database lacks detailed information on chemotherapy, targeted therapy, or immunotherapy, which may affect the prognosis. Fourth, several important variables are not available in SEER database, such as the performance status, comorbidities, and socioeconomic environment, etc. The status of site-specific metastasis information was displayed as "Yes/No/Unknown”, further detailed information was not available. Therefore, interpretation of data is limited and further research is warranted. Fifth, most patients included in this study were white, so caution should be used when applied the results to other ethnic populations.

## Conclusions

In conclusion, among patients with metastatic GC, when the age cutoff value was set at 40 years, the younger patients exhibited more aggressive tumors but significantly better survival outcomes than older patients. In addition, the metastasis patterns, and its effect on the prognosis of GC in different age groups are varied. Compared with the older patients, the younger patients were more prone to having single bone metastasis but less likely to have single liver metastasis. Liver-only metastasis showed the best prognosis in both younger and older patients, while lung-only metastasis showed a worse prognosis in the younger group. These findings highlight the need for increased awareness of the metastatic GC in young patients. Further research investigating molecular changes associated with age-related variations in metastatic patterns could provide deeper insights into disease management.

## Supporting information

S1 Fig Flowchart of patient selection from the SEER database. (TIF)
