# Supplementary figures and images for: Metastasis patterns and prognosis in young gastric cancer patients: A propensity score‑matched SEER database analysis
Source: PLoS One. 2024 Apr 9;19(4):e0301834. doi: 10.1371/journal.pone.0301834 (PMC11003629; doi:10.1371/journal.pone.0301834)

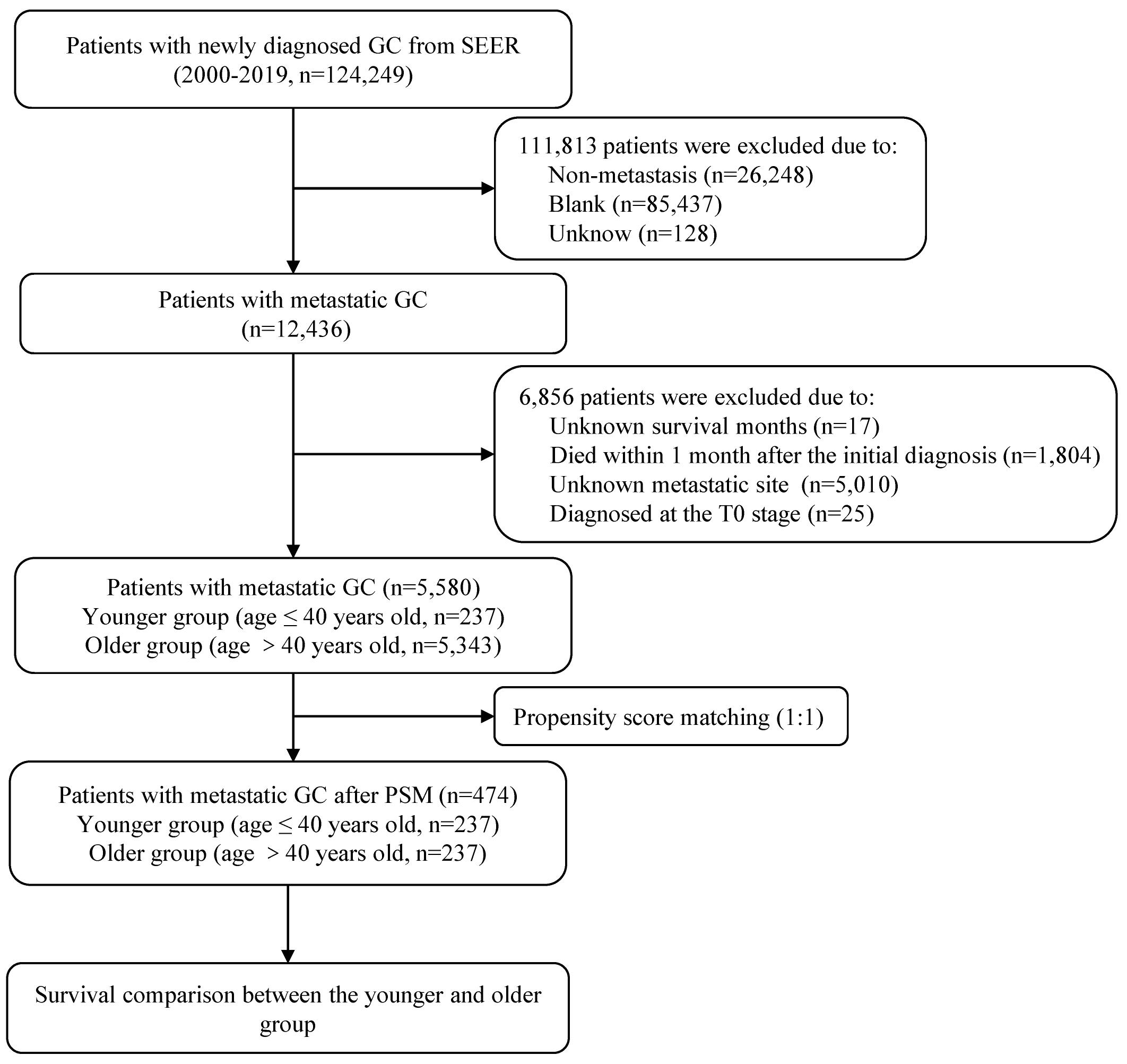

Supplement: S1 Fig — (TIF) [file pone.0301834.s001.tif]
